# Potential Benefits of Non-Pharmacological Therapies in Fibromyalgia

**DOI:** 10.2174/1874312900802010001

**Published:** 2008-01-24

**Authors:** F. Sueiro Blanco, I. Estévez Schwarz, C. Ayán, JM. Cancela, V. Martín

**Affiliations:** 1 Superior Polytechnical School of Lugo, University of Santiago de Compostela, Spain; 2 University of Sport’ Science, Pontevedra, Spain; 3 School of Health and Science, University of Leon, Spain

**Keywords:** Fibromyalgia, therapy, management.

## Abstract

Fibromyalgia (FM) is an incurable common syndrome of non-articular origin, and with no effective treatment by now. A great deal of research has sought to assess the efficacy of different therapies, especially non-pharmacological and low-cost ones, in the reduction of the intensity of symptoms. Despite the availability of a wide range of alternative therapies nowadays, there is little scientific evidence of the potential benefits of most of them, with results being contradictories. The purpose of this paper is to review some of the less well known alternative therapies in FM treatment, to describe the more relevant clinical studies published in this matter, and to analyze the potential effects of the main alternative therapies, in order to verify their efficacy.

## INTRODUCTION

Fibromyalgia (FM) is an idiopathic pain disorder which has been associated with a state of pain amplification and psychological distress [1]. A heterogeneous series of disturbances, mainly involving autonomic, neuroendocrine and neuropsychic systems, is usually present, alongside with symptoms such as sleep disturbance, fatigue, pain, daily function impairment and often stress [2]. The lack of peripheral abnormalities in this disease has led clinicians and researchers alike to question if this syndrome represents a valid entity [3]. However, Harris *et al. *[4] have suggested that FM is a central nervous system disorder, since FM patients have abnormalities within central brain structures that normally encode pain sensations, leading to a hypersensitivity to pain. For these authors, FM patients do not process the body's natural pain relievers efficiently, which it may be due to a dysfunction in their analgesic (painkilling) mechanism.

Current treatments for FM include medical, self-management and alternative interventions. Although the number of published studies has risen steadily over the past decade, treatment remains inadequate to reliably resolve persistent symptoms and improve functional limitations and quality of life in most patients [5]. Pharmacotherapy (specially antidepressants), and nonpharmacologic interventions (mainly exercise, occupational therapy and psychological strategies) have shown moderate evidence for efficacy in this regard [6-8]. However, despite the fact that several alternative and complementary therapies (nonmedicinal ones) have shown little clinical benefit [6], 60%-90% of the patients with FM in the United States have reported to have used one or more complementary or alternative treatments in their disease [9] which states the need for further evaluation of this kind of interventions.

The purpose of this paper is to review some of the more significant clinical studies published in this matter, and to analyze the potential effects of the main alternative therapies in order to verify their efficiency.

## NUTRITION AND DIETS

In chronic diseases like FM, it is quite common that prescribed pharmacologic treatments, alongside with some characteristic symptoms, such as pain, insomnia, fatigue, and depression mood, lead to appetite loss and unhealthy nutritional habits. Because of that, some studies have tested the effects of different nutritional diets, in order to determine their impact on FM patients.

Success with vegetarian diet treatment possible relies on the existence of antioxidants, low fat and protein contents, and high levels of fiber, vitamins (C, and beta-carotene) and minerals (Magnesium, Potassium, Zinc, Selenium), which could attenuate some FM symptoms. Donaldson *et al*. [10] designed a non randomized dietary intervention in order to assess their effects in a 30 FM patients group. A vegetarian diet using raw products like fresh fruit, salads, vegetables, carrot juice, nuts, seeds, whole grain products, tubers, flax seed oil and and virgin olive oil were proposed for 7 months. On the other hand, alcohol, caffeine, refined sugar, corn syrup, refined or hydrogenated oil, refined flour, dairy products, eggs or any type of meat were avoided.

The selected diet were divided in fat (24%), carbohydrates (65%) and proteins (11%) and combined with an intake of 52 mg/day of beta-carotenes due to the carrot juice ingestion. Once the trial was completed, most of the subjects responded favourably, obtaining significant improvements in their quality of life, healthy well being, mobility, flexibility and pain perception.

Kaartinen *et al*. [11] carried out a non-randomized controlled study during 3 months in a 18 fibromyalgia patients group applying a strict vegetarian diet with raw food and low salt content. Fifteen patients as control group continued their omnivorous diet. As soon as the study was over, significant improvements were observed regarding to pain, morning stiffness, sleep and subjective health perception. Other remarkable findings were a reduction of sodium concentration in urine and lower levels of cholesterol in sanguineous serum.

Another similar study [12] evaluated the effects of a raw food diet, mainly based on berries, fruits, vegetables, roots, nuts, germinated seeds and shoots in FM patients. These foods were chosen because of their content of carotenoids like beta and alpha carotenes, lycopene and lutein. An increase in carotenoid levels in sanguineous serum was observed in these patients. The effect of the diet was reflected in a diminution of morning stiffnes and pain and in an increase of their self-experienced health. However, the beneficial effects on the symptoms were temporal and disappeared once the study ended.

Based on the assumption that tryptophan (protein that helps to secrete serotonin in the brain) was low in FM patients and knowing that the intake of protein rich in large neutral amino acids is reported to lower brain tryptophan, Azad *et al*. [13] undertook an open, randomized controlled trial to assess whether any reduction of such proteins by exclusion of animal protein from the diet reduced pain and morbidity in FM patients. A group of 30 FM patients were enrolled in a vegetarian diet with low concentrations of these proteins for 6 weeks, while a 41 patient control group was treated with an antidepressant (amitriptyline) that directly acts on pain perception and insomnia. At the end of the study, the amitriptyline group showed significant improvements in fatigue, insomnia, sleep quality, pain intensity and tender point count; while the vegetarian group only showed a significant but smaller improvement in the pain score.

Another relevant matter is the effect of some diets on the internal microflora. For instance, Hostmark *et al*. [14] analyzed the influence of a 3-week vegetarian diet and fasting on serum concentration of peroxides, lipids, apolipoproteins, and plasma fibrinogen in 10 fibromyalgia/fibrositis patients and found an improvement in the self-experienced perception of health. No control group was used. This positive effect could be due to the peroxide concentration in sanguineous serum and the reduction of some lipoproteins of the total cholesterol.

In another similar non randomized study [15], 35 FM patients received a mostly vegetarian Mediterranean diet or participated in an intermittent modified fasting therapy during 8 days. The first group was enrolled in a normocaloric Mediterranean diet 8372 kJ (2000 kcal/day) which consisted of 7 daily portions of fruit and vegetables, abundant whole grain bread, paste, rice and two portions of fish per week, using only olive oil and canola oil on food preparation.

The other group received 3350 kj (800 kcal/day) of a low salt and calorie diet, with cooked rice and vegetal during two previous days. After three days, patients fasted until the midday of the eleventh day, and then underwent two days of a low calorie diet. From afternoon of 13^th^ the diet was normocaloric. During the fasting days, participants could have tea (nonstimulating), fruit juice and small amounts of light vegetable soup with a maxima of 1255 kj (300 kcal/day), as well as between 2 and 3 litres of daily liquids. Caffeine or alcohol were completely forbidden. Once the trial concluded, there was a reduction in the severity of the symptoms especially in the fasting group. The quantification of the microbiology of the faecal aerobic flora and the anaerobic bacteria (clostridia and bifidobacteria) and candida did not show significant differences between the initial and final data, neither between the values of both treatments. The analysis of the correlation between the course of the disease and the concentration of inmunoglobuline A (sIgA), substance that improves the immune functions, or stool pH did not show any correlation. However, these results contradict Beer *et al*. findings [16], who found a significant increment of sIgA levels (which means an improvement of the immune condition of the intestine and consequently of the whole organism) in 55 chronic pain patients after having participated in a heal-fasting therapy during 3 weeks.

## ACUPUNCTURE

Acupuncture (therapy which is able to reduce pain by means of needles insertion into specified points of the body coincident with nerve endings) is, without a doubt, the treatment with the greatest acceptance within the complementary therapies for the treatment of FM and the most studied. Thus, 22% of the patients with FM declare to have used this treatment [17].

In a clinical randomized trial, Assefi *et al*. [18] studied the effects of acupuncture on 100 patients suffering from FM. Four groups were formed but only in one of them the specific points of acupuncture for this pathology were used. In the other groups, non-specific FM points, treatment points no recognized by habitual practice of acupuncture and a treatment without effective penetration of the needles, were respectively used.

The groups were treated for 12 weeks with a frequency of two 30 minute sessions per week (24 sessions total). Once the treatment ended, no significant differences were observed in pain, fatigue, sleep or general well-being, in any groups.

In the same way, Leibing *et al*. [19] showed that the traditional acupuncture treatments aimed to reduce chronic pain in FM had the same effectiveness that the simulated ones. They carried out a randomized, blinded, placebo-controlled trial study, where 131 FM patients were distributed in three groups during 12 weeks. The control group (n=46) received no further treatment, the acupuncture group (n=40) received 20 sessions of traditional acupuncture and the sham-acupuncture group (n=45) 20 sessions of simulated acupuncture. Once the trial was over, a significant reduction of anxiety and pain was observed in both acupuncture groups, although it was more significant in the traditional one.

Neither difference between simulated or conventional acupuncture were found by Goddard *et al*. [20] in a group of patients suffering from muscular pain. Although a reduction in pain level was observed, it might be due to the mechanical stimulation of the masseter muscles in myofascial pain patients, instead of being related to the location of the stimulus as predicted by the classical acupuncture.

On the other hand, Harris *et al*. [21] carried out a study where 114 FM patients were randomized to one of four treatment groups, combining traditional and not traditional stimulation, with presence or absence of manual needle stimualtion. All groups received treatment once a week, followed by twice weekly, and finally three times weekly, for a total of 18 treatments. Each increase in frequency was separated by a 2-week washout period. At the end of the treatment phase, it was observed that 25%-35% of the patients had experienced an overall improvement in pain; however this was not related to any of the specific treatment proposed.

Finally, it is important to note that after reviewing some clinical studies, Ezzo *et al*. [22] concluded that there is limited evidence that acupuncture is more effective than placebo, sham acupuncture, standar care or no treatment, for patients suffering from chronic pain.

## ELECTROACUPUNCTURE

Electroacupuncture (EA), variation of conventional acupuncture consisting on applying electrified needles (with a maximum intensity of 10 mA) through the skin, has been regarded as useful non-pharmacological therapy. In this regard, Deluze *et al*. [23] examined their effectiveness in a randomized study applying EA therapy to a group of 36 FM patients, whereas another similar group of 34 patients was treated with placebo treatment similar to conventional acupuncture. The participants received 6 sessions for three weeks. The EA group received treatment in two peripheral points of acupuncture and 6 zones near tender points (TP), applying a light discharge, enough to produce muscular contraction. The first group improved considerably in all aspects (pain threshold, pain scales, sleep quality and patient’s medical appreciation) except morning stiffness and regional pain.

In another randomized clinical trial, Guo and Jia [24] treated different groups of FM patients with electroacupuncture therapy (n=22), another therapy based on dermo-neurological electrical stimulation (DEA) (n=22) and one third (n=22) providing endorphin stimulation (oryzanol) during 45 days. At the end of the treatment, no significant differences were observed in the symptoms studied between the group of EA and DEA, although a nonsignificant improvement in the first was found. However, important differences were observed between these two treatments and the third with oryzanol.

## HYDROTHERAPY

The traditionally known utility of water, mainly based on some of its characteristics (temperature, mineral content, etc), in some rheumatic conditions, has raised the question whether FM patients could benefit from different modalities of hydrotherapy. In this regard, many authors affirm that the balneotherapy (or tempered water bath) seems to be effective with the muscular pain [25]. This could be due to the fact that the muscular tone, joint mobility and pain intensity are affected by the thermal and hydromechanical stimuli, which cause an analgesia in the nerve endings that increases the pain threshold, easing the muscular spasms [26]. Moreover, a peripheral vasodilatation takes place, as well as an increase in the tendon extensibility, which benefits the conjunctive tissues. This helps to improve muscular isquemia and reduce the algesic mediators in FM [27]. Balneotherapy also seems to activate the parasimpatic system, increasing the accumulation of acetylcholine in central nervous system and causing a sedative effect factor [28]. On the other hand, some authors think that balneotherapy produces beta-endorphin liberation [29], but some studies in rheumatic patients indicate the opposite [30].

Another prophylactic property of hydrotherapy is based on the concentration of elements and mineral compounds like Carbon Dioxide, Calcium, Magnesium, Lithium and Sulphate, dissolved in water and that can be absorbed through the skin, providing healing effects in several body organs and circulatory system, reducing muscular spasms and pain sensitivity and increasing joint mobility, as well as peripheral circulation [31].

Ardiç *et al*. [32] evaluated in a randomized study the influence of balneotherapy on inflammatory mediators, applying a therapy of 20 minute baths, 5 days a week during 3 weeks with water at 36ºC. Once the whole therapy ended, there were significant improvements in pain and depression levels and in the impact of the disease in treatment group (n=12), with respect to the control group (n=12). In this study a significant reduction in the sanguineous serum of inflammatory markers IL-1 and LTB4 and a smaller one in PGE2, was observed. Since these substances were detected in the skin of some FM patient [33] and were strongly related to the inflammatory processes that cause the pain sensation [34], their reduction after the treatment could indicate a positive effect.

Physical exercise has been shown to be a useful tool in the treatment of FM. Altan *et al*. [25] compared in a randomized study the effect of pool-based exercise on 25 FM patients versus a 25 subjects control group which received balneotherapy consisting on baths of 35 minutes three times a week, during 12 weeks in a swimming pool with water at 37ºC. Both therapies showed significant improvements in pain, fatigue, morning stiffness, quality of sleep, health perception, functional capacity and physical and global well-being, which lasted for 12 weeks. It must be emphasized that pool-based exercise had a longer-lasting effect on some symptoms, but the study did not show that physical exercise were more effective than balneotherapy alone.

The influence of the chemical composition and the temperature of the water on the balneotherapy has also been evaluated. Thus, Evcik *et al*. [35] used natural spring water to observe their effects in FM in a non randomized study. The water had the following composition: Sodium (278 mg/L), Bicarbonate (677 mg/L), and Sulphate (96 mg/L) principally, and also Calcium, Magnesium, Iron, Aluminium, Chlorine anions and Metasilicate. A group of 22 patients received balneotherapy treatment, consisting of a daily bath of 20 minutes, 5 times a week with a temperature of 36ºC for 3 weeks, whereas the control group (n=20) did not receive any treatment. The results showed an improvement in health perception, functional capacity, and TP, which were maintained for 6 months. In another similar randomized study, Ammer *et al*. [36] studied the effect of different types of baths, like plain waters (n=11), waters with pine oil (n=7) or with valerian (n=12) on pain, quality of sleep and TP on FM patients. The baths were carried out in a total of 10 sessions, three times a week. The patients in the valerian group registered significant improvements in all of symptoms; while subjects treated with pine oil water improved their well-being but pain threshold did not diminish practically. Finally, the baths with clear water reduced significantly the general pain and their intensity.

Another important issue in hydrotherapy is the possible beneficial effect on the well-being of FM patients, after staying in therapeutic resorts surrounded by a relaxed and therapeutic atmosphere. In this regard, other types of hydrotherapy, like the ones which take advantage of the properties of some marine waters (Thalassotherapy) or those that combine diverse therapies in some tourist resorts (Spa), have been also reviewed.

Thus, Neumann *et al*. [37] studied in a non randomized trial the potential benefits of “Spa” treatment, at the Dead Sea. One group of FM patients (n=24) underwent balneotherapy sessions of 30 minutes in sulphurous swimming pools at 37ºC for 10 days, whereas the control group (n=24) did not receive any treatment. Once the therapy ended, a significant improvement in quality of life, pain and fatigue was registered. This improvement was greater in the balneotherapy group and lasted longer. Similar results (specially improvements in the physical and functional condition, as well as a reduction in the number of TP) were observed in different studies, which recalled the effectiveness of balneotherapy on patients with fibromyalgia at the Dead Sea [38,39].

Finally, beneficial effects of Thalassotherapy in FM patient’s quality of life have been reported too [40, 41], which imply that hydrotherapy shows favourable cost-effectiveness and cost-utility ratios compared with FM standard treatments.

## BALNEOTHERAPY AND ELECTRICAL STIMULATION

Hydroelectric baths have been used as an effective way of treatment some rheumatic disease, for instance the “Stanger bath”, where low frequency currents (galvanic or diadynamic) are given to the patient, in order to reduce acute pain and muscular disturbances [42]. In a randomized study, Eksioglu *et al*. [43] treated a group of 50 FM patients with amitriptyline (most commonly for FM treatment) in a dosage of 10 mg daily combined with 20 minute daily sessions of "Stanger bath" in warm water (37ºC) for a total of ten sessions distributed in a period of two weeks. Another group received only pharmacologic treatment. The combined treatment showed significant improvements in TP score, quality of life and global functional capacity, obtaining in addition a more lasting effect.

The effectiveness of hydrogalvanic therapy has been also compared to relaxation techniques. In this regard, twelve patients underwent hydrogalvanic baths while 13 patients received a Jacobson relaxation training [44], but apart from one difference in the different dimensions of pain measured in the beginning and the end of therapy, no differences between the two treatments were found.

## MAGNETOTHERAPY

The treatment of FM symptoms using static or pulsed magnet fields, attached to the surface of the body has been also evaluated. Magnetotherapy can produce positive changes in the immunological condition of the patient, such as vasodilatation of the arterial part of capillaries and analgesic and anti-inflammatory action. In this regard, Lena and Friol *et al*. [45] carried out a non randomized study with two groups of 25 FM patients each. One of them underwent a pharmacologic conventional treatment for 10 days. The second group completed 10 sessions of magnetotherapy using a magnetic bed, with solenoid placed in the dorsal region of the patient in prone position. At the end of the treatment, both groups reduced their TP score, with the effect lasting for 25 days. However, a great improvement was observed in the pharmacological group.

In a similar randomized study, Alfano *et al*. [46] carried out a 6 months’ research with five groups of FM sufferers. The first group used a pad that provided whole-body exposure to a low, uniform static magnetic field of negative polarity (CMU), while the second group used a pad that exposed them to a low static magnetic field that varied spatially and in polarity (CMV). Two groups were given a placebo treatment using inactive magnets and finally a control group with a conventional treatment were established. Once the trial ended, the functional pad groups showed improvements in functional status, pain intensity level, tender point count, and tender point intensity. With the exception of pain intensity level these improvements did not differ significantly from changes in the Sham group or in the Usual Care group.

Finally, Colbert *et al*. [47] in a randomized study with 35 subjects analyzed the effect of sleeping on a magnetic mattress pad and found significant decreases in pain, fatigue and total myalgic score during the 16 weeks that the study lasted. Although another group who slept on a sham mattress pad experienced no significant change in the previous measures, the placebo effect could be observed on tiredness and well-being wakening improvement.

## HYPERBARIC OXYGEN

The breathing of pure oxygen in a sealed chamber was proposed for the treatment of FM by Fassbender *et al*. [48]. According to these authors, a high level of oxygen concentration, combined with higher oxygen partial pressure, could fight the possible local hypoxia related to the disease aetiology, since low oxygen pressures have been found in muscular and subcutaneous tissue [49]. In this regard, Yildiz *et al*. [50] carried out a randomized study exposing a group of 26 FM patients to 90 minute daily sessions of hyperbaric treatment at 2.4 atmospheres, five days a week during three weeks. A second group (n=24) received air at 1 atmosphere and acted as control group. After receiving treatment, the hyperbaric group showed a significant improvement in TP scores, pain threshold and pain severity. On the other hand, significant differences between both groups for all parameters were also registered, except for the pain severity after the first session.

## ULTRASONOGRAPHY

Ultrasound by means of high electromagnetic frequency or acoustic waves, have been effective in the treatment of myofascial pain [51], mainly due to the effect of increasing blood flow [52], membrane permeability [53] and capillary density in skeletal muscles [54].

Gonzalez-Viejo *et al*. [55] decided to assess their effectiveness in the treatment of FM symptoms in a randomized study. Two FM groups underwent two different therapies for 6 months. The first group (n=36) was treated daily with an antidepressant (sertraline), with doses of 50 mg/day; whereas the second group (n=34) experimented 15 sessions of 1 W/cm2 of ultrasonography on the cervical trigger points plus physical therapy. The results of this study showed that patients treated with sertraline had better outcome in terms of pain, morning stiffness and sleep disorders. Regarding patient perception, 6% of patients treated with ultrasonography perceived the treatment as good therapy for their health, while 83% of patients treated with sertraline felt the same. In a similar study, Almeida *et al*. [56] assessed the effects of combined therapy with pulsed ultrasound and interferential current on pain and sleep in 17 FM patients. The treatment group received 12 sessions of the combined therapy for 4 weeks, including 4 nights sleeping in a dream laboratory. The control group received a simulated treatment using an inactive system. At the end of the study, the first group showed a subjective improvement in pain (number and intensity of painful areas) as well as an objective improvement (TP scores). On the other hand, subjective sleep improvements, and reduced morning fatigue, were observed too. This result recalls the effectiveness of combining ultrasound and interferential current, in treating pain and sleep manifestations in FM patients.

## CONCLUSION

Although it seems feasible to mitigate the FM symptoms by means of applying some of the therapies commented here, the number, the intensity and the short duration of the beneficial effects are not as powerful so as to overcome the traditional therapies. On the other hand, the fact that in some of the clinical trials, the control or sham therapies obtained similar positive results reveals the special importance of the placebo effect in this type of studies. Because of this, far better controlled trials are needed, in order to determine the real efficacy of the alternative, non-pharmacological approach in FM.
